# Magnesium-Doped Hydroxyapatite Nanofibers for Medicine Applications: Characterization, Antimicrobial Activity, and Cytotoxicity Study

**DOI:** 10.3390/ijms252212418

**Published:** 2024-11-19

**Authors:** Ricardo Pascual Alanis-Gómez, Fabiola Hernández-Rosas, Juan David Olivares-Hernández, Eric Mauricio Rivera-Muñoz, Araceli Zapatero-Gutiérrez, Néstor Méndez-Lozano, José Rafael Alanis-Gómez, Rodrigo Velázquez-Castillo

**Affiliations:** 1 División de Investigación y Posgrado, Facultad de Ingeniería, Universidad Autónoma de Querétaro, Querétaro 76010, Mexico; ralanis17@uaq.mx; 2 Escuela de Ingeniería Biomédica, División de Ingeniería, Universidad Anáhuac Querétaro, Querétaro 76246, Mexico; fabiola.hernandezro@anahuac.mx; 3 Centro de Investigación, Universidad Anáhuac Querétaro, Querétaro 76246, Mexico; araceli.zapatero@anahuac.mx; 4 Facultad de Química, Universidad Autónoma de Querétaro, Querétaro 76010, Mexico; 5 Escuela de Terapia Física, Universidad Politécnica de Santa Rosa Jáuregui, Querétaro 76220, Mexico; jolivares@upsrj.edu.mx; 6 Centro de Física Aplicada y Tecnología Avanzada, Universidad Nacional Autónoma de México, A.P.1-1010, Querétaro 76010, Mexico; emrivera@fata.unam.mx; 7 Ingeniería Mecánica para la Innovación, División de Ingenierías, Universidad Anáhuac Querétaro, Querétaro 76246, Mexico; 8 Departamento de Ingeniería, Universidad del Valle de México, Campus Querétaro. Blvd. Juriquilla No. 1000 A, Del. Santa Rosa Jáuregui, Querétaro 76230, Mexico; nestor.mendez@uvmnet.edu

**Keywords:** magnesium-doped hydroxyapatite, biocompatibility, cytotoxicity, antimicrobial activity

## Abstract

Magnesium-doped hydroxyapatite (HAp-Mg) nanofibers show promise for medical applications due to their structural similarity to bone minerals and enhanced biological properties, such as improved biocompatibility and antimicrobial activity. This study synthesized HAp-Mg nanofibers using a microwave-assisted hydrothermal method (MAHM) to evaluate their cytotoxicity, biocompatibility, and antimicrobial efficacy compared to commercial hydroxyapatite (HAp). Characterization through X-ray diffraction (XRD), scanning electron microscopy (SEM), Transmission Electron Microscopy (TEM), energy-dispersive X-ray spectroscopy (EDS), and Fourier transform infrared spectroscopy (FTIR) confirmed the successful incorporation of magnesium, producing high-purity, crystalline nanofibers with hexagonal morphology. Rietveld refinement showed slight lattice parameter shortening, indicating Mg^2+^ ion integration. Cell viability assays (MTT and AlamarBlue) revealed a significant increase in fibroblast proliferation with 2% and 5% HAp-Mg concentrations compared to controls (*p* < 0.05), demonstrating non-cytotoxicity and enhanced biocompatibility. Antimicrobial tests (disk diffusion method, 100 µg/mL) showed that HAp-Mg had strong antibacterial effects against Gram-positive and Gram-negative bacteria and moderate antifungal activity against *Candida albicans*. In contrast, commercial HAp showed no antimicrobial effects. These results suggest HAp-Mg nanofibers have significant advantages as biomaterials for medical applications, particularly in preventing implant-related infections and supporting further clinical development.

## 1. Introduction

For the past few years, bone regeneration has become an important focus in the field of biomaterial research. One promising approach has been the use of nanostructured materials that have a structure and chemical composition like that of bone. The case of hydroxyapatite (HAp) is a biocompatible and bioactive ceramic nanocomposite that is widely used in tissue engineering due to its similarity with the mineral component of bone [1]. In addition, synthetic HAp has been the most stable calcium phosphate in vitro biological studies because it is non-toxic, causes minimal inflammatory response, and is osteoconductive [2,3,4,5].

The biocompatibility of synthetic HAp has been previously investigated in different studies [6,7,8,9,10]. Lin et al. (2009) reported that HAp is biocompatible and promotes the osteoinduction and osteogenic differentiation of mouse stem cells [10]. Jing et al. (2021) evaluated the cytotoxicity and biocompatibility of a biofunctional HAp gradient coating on MC3T3-E1 osteoblasts using an in vitro cell proliferation assay. In general, the tests demonstrated that the HAp-based coating was not cytotoxic and was favorable for osteoblast proliferation, facilitating a uniform cell distribution on the biomaterial [6]. Due to these factors, HAp has emerged as a viable alternative in the field of bone regeneration, being a particularly useful alternative to autografts. However, the low mechanical strength of HAp limits its application as a load-bearing implant. Doping HAp with metal ions has been proposed to improve its mechanical properties and biological performance. The use of bioactive and biocompatible materials, such as magnesium-doped HAp nanofibers (HAp-Mg), has been shown to be a promising strategy for enhancing bone regeneration [11]. Magnesium is an essential element for bone and teeth formation and maintenance, and its presence in hydroxyapatite can improve osteoblastic activity and osteointegration [12].

Several studies have explored the synthesis and properties of magnesium-doped HAp nanofibers for use in bone regeneration. For example, Iconaru et al. (2022) aimed a study to develop and evaluate the biological and physicochemical properties of magnesium-doped HAp in a chitosan matrix composite coating as well as to investigate the influence of magnesium concentration on these properties. The results showed that the incorporation of magnesium into the HAp structure improved its mechanical properties and bioactivity, making it a promising material for bone regeneration applications. The study also found that the composite coatings exhibited good biocompatibility and promoted cell adhesion and proliferation [13].

Another study conducted by Predoi et al. (2022) reported the evaluation of a vacuum deposition technique that was used to create composite thin films based on chitosan-coated HAp-Mg, and it was found to be effective in biological assays. The samples were exposed to gamma irradiation doses, but this did not result in significantly higher rates of death of fibroblast in culture [14]. Moreover, the appearance of pores on the surface of the coatings after gamma irradiation did not affect the adhesion and cell spreading. Therefore, these thin films may be suitable for use in bone implants and other orthopedic and dental applications [14]. In addition, HAp-Mg nanofibers have also demonstrated antimicrobial properties [14,15,16]. Finally, the synthesis and characterization of HAp-Mg nanofibers have shown promising results for bone regeneration and preventing infections. However, it is essential to evaluate the cytotoxicity of these materials to ensure their safety and clinical efficacy.

In this work, a facile and cost-effective synthesis method was used to synthesize pure and magnesium-doped HAp. Antimicrobial exams demonstrated that the HAp nanofibers showed antibacterial and antifungal activities, and commercial HAp did not. Crystallinity, the morphology of fiber, and the preferential crystalline orientation were relevant factors in improving this antimicrobial activity. Additionally, the inclusion of magnesium ions in the crystal structure of HAp nanofibers significantly increased this antimicrobial activity.

## 2. Results

### 2.1. Characterization of HAp-Mg Nanofibers

#### 2.1.1. Scanning Electron Microscopy

The morphological and topological characterization of pure (commercial and nanofiber) HAp and magnesium-doped HAp (HAp-Mg 2% and HAp-Mg 5%) was performed using scanning electron microscopy (SEM). The samples (except for the commercial one) exhibited a well-defined morphology of fiber and a uniform hexagonal cross-section.

**Figure 1:**
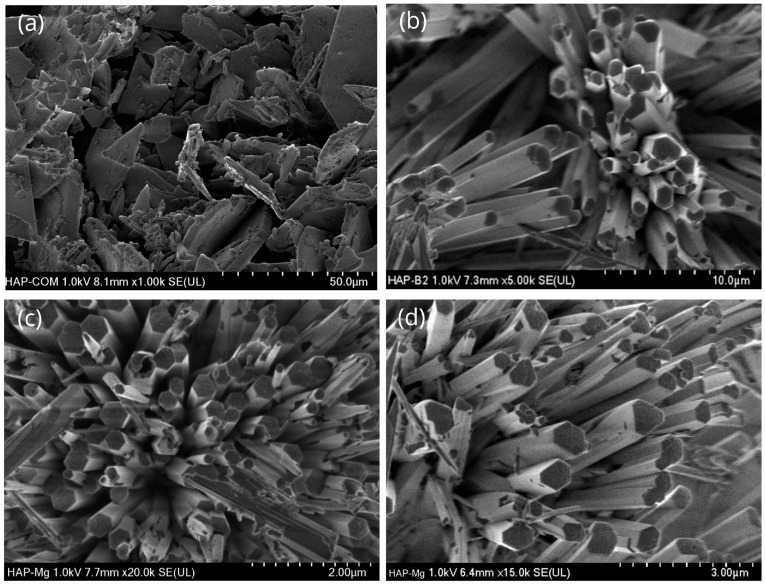
Comparative morphology analysis of different HAp aggregates. Commercial HAp (**a**), pure HAp obtained with MAHM (**b**), HAp-Mg 2% (**c**), and HAp-Mg 5% (**d**). The last two were also synthesized using MAHM.

The morphological analysis of commercial HAp shows irregular shape aggregates and a dispersed particle size distribution; most of those aggregates have an average particle size of less than 200 nm (Figure 1a). This morphology is a common result of the random crystal growth.

In contrast, the pure HAp synthesized using the MAHM presents a markedly different morphology (Figure 1b). This HAp shows a highly ordered morphology of fibers. These crystalline structures are tightly packed, forming conglomerates reminiscent of orderly resembling pencils. All external surfaces on the fibers appear smooth and uniform, lacking conspicuous irregularities. This morphology is the result of using the MAHM to obtain the structures and using glutamic acid to guide the crystal growth.

In the case of the magnesium-doped one, a morphology like that observed for pure HAp fibers was found. In Figure 1c,d, the well-defined ends of these fibers suggest consistent and reproducible growth throughout the synthesis process. According to Figure 1b–d, the addition of Mg^2+^ doping ions did not significantly modify the morphology of fibers.

**Figure 2:**
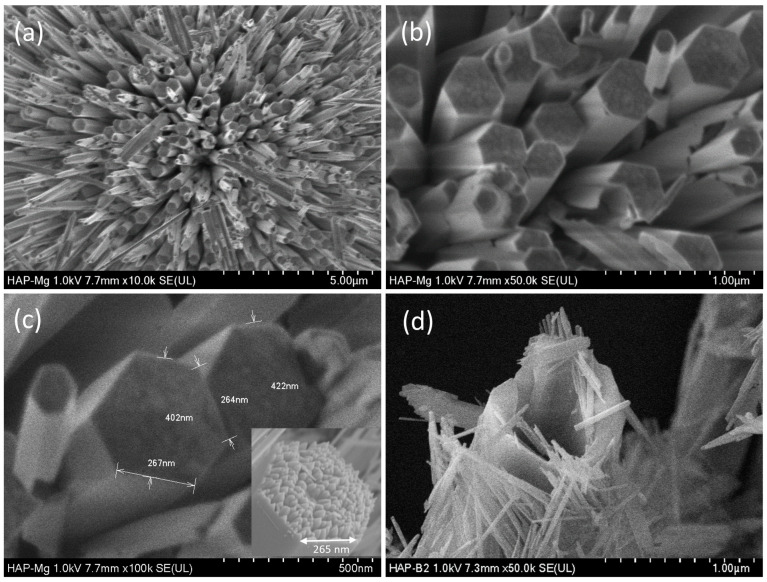
SEM micrographs of HAp-Mg 2% sample, (**a**) showing the microfiber arrangement, (**b**) viewing the hexagonal cross-section of microfibers, and (**c**) displaying different microfiber dimensions. The inset shows how the microfibers are made by the union of several nanofibers. (**d**) Shows the nanofibers that were contained in a microfiber.

A deeper observation of the HAp-Mg 2% sample corroborates the morphology of rather organized nanofibers (Figure 2a), and the examinations also allowed us to see smooth lateral surfaces and well-defined ends (Figure 2b,c). The hexagonal cross-section is not always regular. Its average separation of parallel sides was 402 nm, and its length was found to be in the range of 20–30 µm. Size distributions of the parallel sides and length of fibers were narrow and monomodal (especially parallel sides). In the inset shown in Figure 2c, it is possible to observe that the fibers (microfibers) are formed by a compact arrangement of small nanofibers. These nanofibers also have a hexagonal cross-section, and they are united by their lateral facets to form the respective hexagonal cross-section of the microfibers in an interesting hierarchical structural organization. This distinctive arrangement is indicative of a discernible and well-organized geometric pattern.

Some microfibers were thermally treated to be broken and expose their content. Figure 2d depicts a fragmented microfiber and the nanofibers that were released. Dimensions and the hexagonal cross-section of nanofibers could be observed.

#### 2.1.2. X-Ray Diffraction

**Figure 3:**
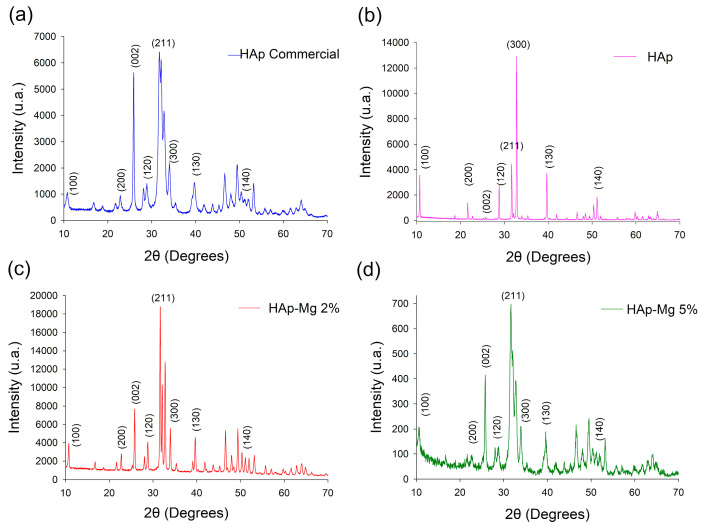
Comparison of XRD diffractograms of the commercial HAp (**a**), HAp made by MAHM (**b**), HAp-Mg 2% (**c**), and HAp-Mg 5% (**d**). Indexing is indicated according to ICDD-JCPDS-PDF #09-432.

The synthesized HAp-Mg 2% and HAp-Mg 5% samples were subjected to analysis via X-ray diffraction by powders (XRD) to identify the crystalline phases present in the materials. These results were then compared with the corresponding diffractograms obtained for the commercial HAp sample and for the pure HAp nanofiber sample obtained using MAHM. The acquired diffractograms are depicted in Figure 3.

It can be observed that the diffractogram of the commercial pure HAp depicted in Figure 3a shows wide and defined Bragg reflections, and the background is quite noisy. The width in the Bragg reflections and the noise in the background are evidence of a low crystallinity in the sample. Crystalline phase identification was made by comparison, and the only phase found was hexagonal HAp according to the ICDD-JCPDS-PDF# 09-0432. As expected, the intensity and angular position of all Bragg reflections coincided with the mentioned PDF.

In contrast, the diffractogram acquired from the pure HAp sample made using MAHM shows quite thin and well-defined Bragg reflections, and the noise in the background is scarce (Figure 3b). These characteristics indicate a high crystalline quality in this material. All reflections have the angular position registered in PDF# 09-0432, but some of them have different intensities than those reported. The reflection at 33° in 2θ, corresponding to (300), has a rather higher intensity than that stated in the said PDF, and the reflection at 10.84° (corresponding to 100) also increased its intensity. Contrarily, the Bragg reflection at 25.88° related to (002) underwent a considerable shortening in intensity, and it is almost null. This variation in the reflection intensities is evidence of a remarkable preferential crystalline orientation.

In the case of the HAp-Mg 2% sample, the diffractogram shows a crystallinity similar to that observed for the pure HAp made through MAHM but is notable in the intensity reduction in the Bragg reflection produced by (300), and this is indicative of a loss in the preferential crystalline orientation (Figure 3c). SEM results indicated that the morphology of nanofiber remains after Mg ion doping, but the entry of these doping ions modified the HAp crystal structure.

Upon increasing the concentration of Mg^2+^ doping ions in the synthetic hydroxyapatite (HAp-Mg 5%), a decrease in crystallinity becomes evident, as reflected in its progressively noisier diffractogram; Bragg reflections also became wider. This deterioration is attributed to the disruption of the crystal lattice caused by the incorporation of Mg ions. Doping ions altered the preferential crystalline orientation and decreased the crystallite size. The diffractogram obtained for the HAp-Mg 5% sample (Figure 3d) is similar to that acquired for the commercial HAp. The identification of the crystalline phases for the HAp-Mg 2% and HAp-Mg 5% were also performed by comparison, and hexagonal HAp was identified according to PDF #09-432.

Crystalline phase identification performed for all samples indicated the presence of only one crystalline constituent. This reveals that the microwave-assisted hydrothermal method is appropriate to synthesize HAp crystals with a high purity.

**Figure 4:**
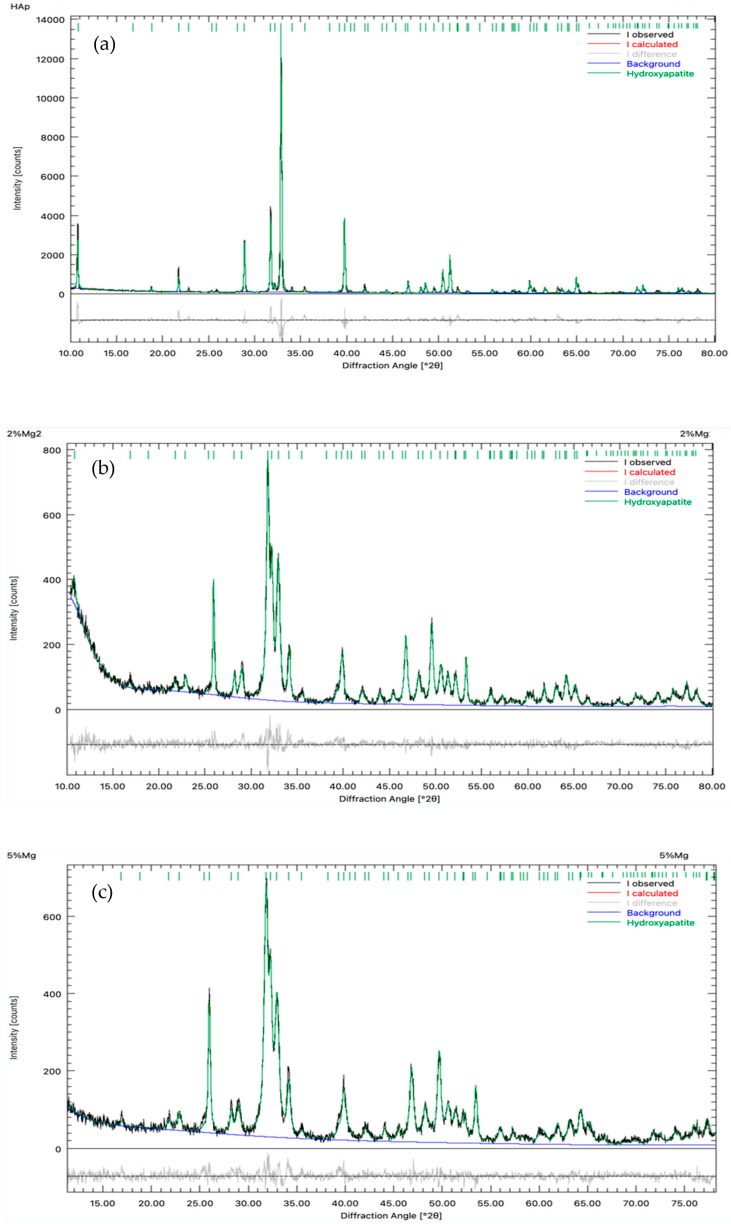
Rietveld refinement of the samples, (**a**) pure HAp, (**b**) HAp-Mg 2% and (**c**) HAp-Mg 5%.

Rietveld refinement results revealed that a slight shortening occurred in the lattice parameters of the doped HAp, and this indicates that Mg^2+^ ions have been incorporated into the HAp crystal structure. Figure 4 shows the refinement carried out for the diffractograms of the pure HAp, HAp-Mg 2%, and HAp-Mg 5% samples. Table 1 displays the lattice parameter values obtained for all samples. Some Ca^2+^ ions (radius 114 pm) were replaced by Mg^2+^ ions (radius 86 pm), and the difference in ionic radius produced a consistent shortening in all lattice parameters of 0.13% when the HAp was doped with 2% wt. of Mg. Mg concentration increased up to 5% wt., but the lattice parameter varied differently. Parameters “a” and “b” were modified by 0.153%, and parameter “c” changed by 0.33%. Crystallite size decreased consistently with the increment of Mg concentration, and according to the diffractograms in Figure 3, crystallinity also reduced with the increase in doping concentration.

The (300) planes form the lateral facets of the fibers, and these planes contain columns made of calcium ions, which are located along the “c” axis of the HAp crystal structure. Therefore, calcium ions are very close to the lateral surfaces of the fibers, and they can be more easily replaced by doping ions. The shortening in the “c” parameter and the intensity reduction in the Bragg reflection corresponding to the (300) that was observed in the diffractograms of the doped HAp samples could be partially explained by an ionic substitution preferentially in the calcium ions of these planes.

#### 2.1.3. Transmission Electron Microscopy

**Figure 5:**
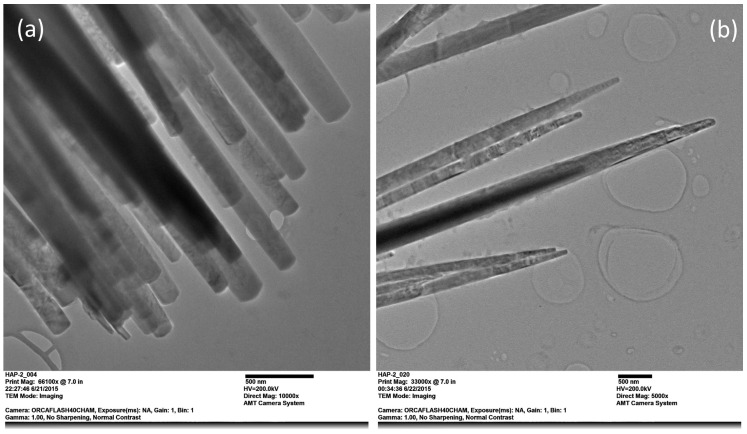
(**a**) TEM micrographs showing the morphology of pure HAp nanofibers and (**b**) HAp-Mg 5%.

Observations performed through TEM confirmed the SEM results; there were no major changes in the morphology of fibers with the addition of dopant. Figure 5 displays the morphology present in the nanofibers with different concentrations of Mg ions. Pure HAp nanofibers have an average thickness of 96 nm, which is rather homogeneous, and their tips are flat (Figure 5a). In the case of the HAp-Mg 5%, thickness decreases toward the end of the nanofibers, and the tips are sharp. The average thickness for this kind of fiber was 89 nm. The greatest differences in the crystal structure of nanofibers were found in X-ray experiments.

#### 2.1.4. Attenuated Total Reflection-Fourier Transform Infrared Spectroscopy (ATR-FTIR)

**Figure 6:**
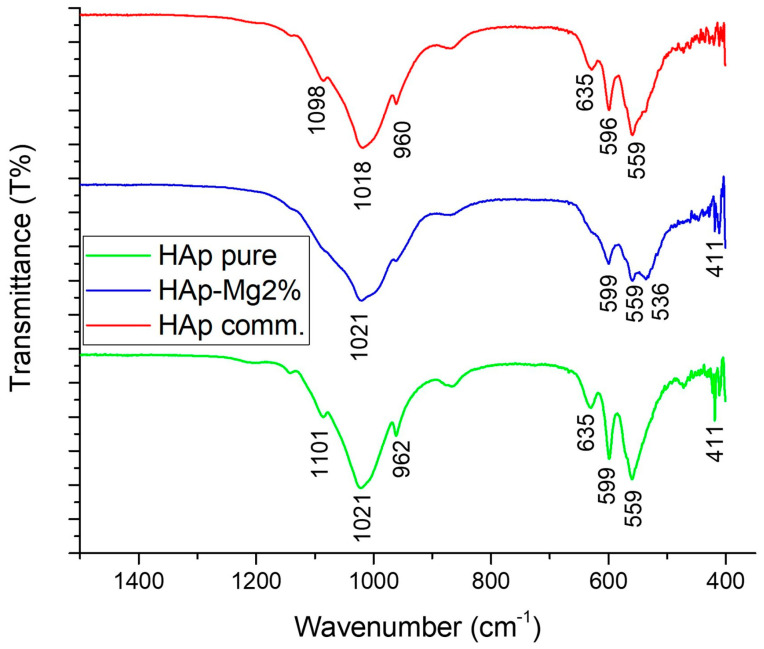
FTIR spectrum of different hydroxyapatites, commercial HAp (HAp comm), HAp-Mg 2%, and pure HAp, both obtained by MAHM, in the spectral window 500–4000 cm^−1^.

FTIR analysis of the spectra of HAp-Mg, pure HAp, and commercial HAp revealed distinctive spectral features in the region of 4000 to 500 cm^−1^, providing valuable information on the structure and composition of the sample (Figure 6; Table 2). An absolute threshold of 74.574 was set for accurate identification of relevant bands. The bands observed in the spectrum of the three HAp samples were recorded in the positions of 536, 559, 599, 635, and 1021 cm ^−1^, each with different intensities that reflect the relative abundance of the functional groups present in the sample. The vibrational assignments of the HAp samples are seen in Table 2 and Figure 6.

The assignment of these bands was made based on the previous literature and complementary analyses. The peak observed at 411 cm^−1^ in the pure HAp sample was associated with the characteristic vibrations of P–O bonds in the HAp phosphate structure due to the vibration of phosphorus and oxygen atoms in the crystal lattice. Its measured intensity of 67,800 indicates a significant abundance of this functional group in the sample (Figure 6).

The prominent bands 1018 cm^−1^ found for commercial HAp correspond to the stretching vibrations of the phosphate bond (PO_4_^3−^) in triply degenerate asymmetric stretching vibration mode, which is generally found in the range of 1020-1090 cm^−1^ [17] (Figure 6). The band observed at 1021 cm^−1^ present in pure HAp and HAp-Mg also corresponds to the vibration of the phosphate group previously mentioned, but this band is slightly shifted to a higher wavenumber. The phosphate groups within nanofibers are located near their surfaces, and the shifting in this signal could be indicative that these groups are freer and can vibrate more easily. The signals at 960 and 1098 cm^−1^ are produced by commercial HAp and correspond to vibrations of the phosphate group. These signals also underwent shifting in the cases of HAp pure and HAp-Mg 2%, where the signals appeared at 962 and 1101 cm^−1^, respectively.

The band at 596 cm^−1^ present in the commercial HAp corresponds to bending vibrations of the phosphate bond (PO_4_^3−^); in particular, it corresponds to the triply degenerate bending mode that is located between 575 and 610 cm^−1^ [17]. This band also underwent a shift to a higher wavenumber (599 cm^−1^) for the samples HAp-Mg 2% and HAp pure for the abovementioned reason.

The presence of magnesium in the doped HAp was evidenced by the band at 536 cm^−1^, corresponding to the vibrations of the Mg–O bonds (Table 2). This band exhibited an intensity of 40.748, indicating substantial incorporation of magnesium ions into the HAp crystal lattice. Furthermore, additional bands were observed in the three HAp samples, such as the band present in 559 cm^−1^ corresponding to the bending or torsion vibrations of the phosphate bond (PO_4_^3−^) with intensities of 68,954, 48,780, and 28,352, respectively, suggesting the presence of these functional groups in the HAp crystal structure (Figure 6; Table 2).

#### 2.1.5. Energy-Dispersive X-Ray Spectroscopy Analysis

**Figure 7:**
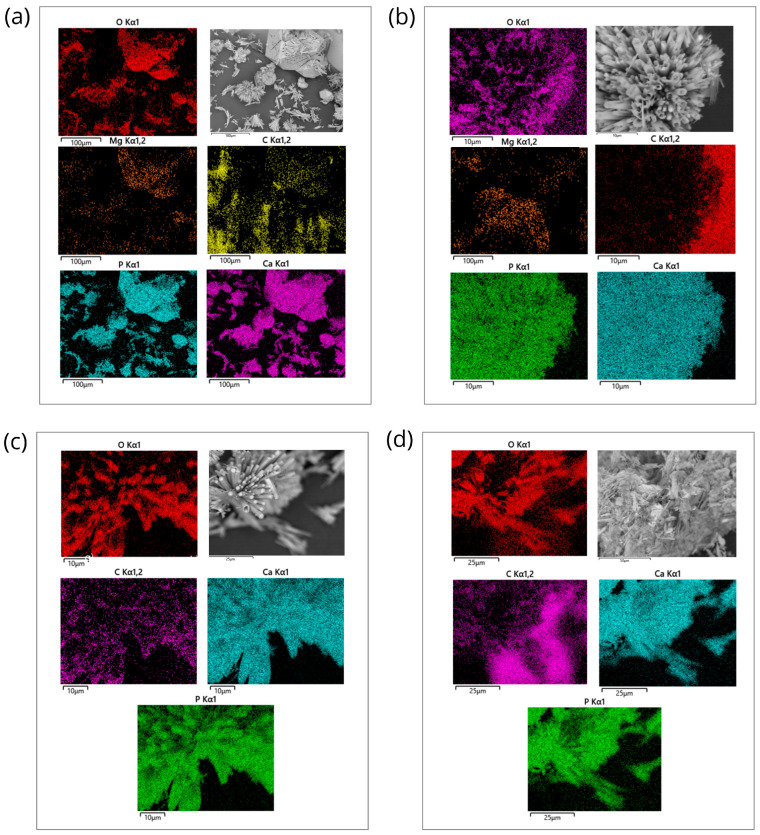
Energy-dispersive X-ray spectroscopy analysis of nanopowder samples of HAp. (**a**) Elemental mapping of HAp-Mg 2% nanofibers in a selected microscopic SEM area, scale bar of 100 µm; (**b**) elemental mapping of HAp-Mg 5% nanofibers in a selected microscopic SEM zone, scale bar of 100 µm; (**c**) elemental mapping of pure HAp synthesized by MAHM in a selected microscopic SEM area, scale bar of 100 µm; and (**d**) elemental mapping of commercial HAp in a selected microscopic SEM zone, scale bar of 100 µm.

The elemental composition of the HAp-Mg 2% samples was obtained by energy-dispersive X-ray spectroscopy analysis. Elemental analysis was performed to further confirm the presence of magnesium in the HAp samples, and uniformity and distribution of doping ions were, therefore, investigated. The EDX analysis of the HAp-Mg 2% nanofibers revealed the presence of calcium, phosphorus, oxygen, and magnesium in the sample. The percentage of magnesium in the sample was found to be 0.3%, indicating the successful incorporation of magnesium ions in the HAp lattice (Figure 7).

Additional analysis was performed by energy-dispersive spectroscopy (EDS) on HAp-Mg 2% and HAp-Mg 5% nanoparticles with Quantax EDS (Bruker Nano GmbH, Germany) for comparative purposes. The results provided important information on their elemental composition and dopant distribution.

This study confirmed the presence of magnesium in the HAp nanofibers, successfully demonstrating the magnesium doping process, as shown in the spectrograms. In addition, the amount of incorporated magnesium was quantified (see Table 3), providing crucial information to control the dopant concentration and tune the properties of the nanoparticles to suit specific applications.

Evaluation of the homogeneity of magnesium distribution from EDS analysis indicated a uniform distribution throughout the samples, suggesting consistency and maintenance of its properties. The elemental composition of the HAp-Mg was calculated using the EDS data (Table 3), which showed a Ca/P ratio of 1.66, in agreement with the stoichiometry of HAp and was likely obtained by the bone tissue.

### 2.2. Biocompatibility, Cell Viability, and Cytotoxicity In Vitro Assays

HAp-Mg has been identified as a promising biomaterial with improved mechanical properties for use in bone regeneration. To assess its biocompatibility and cytotoxicity, primary fibroblast cell cultures were exposed to treatments with HAp-Mg 2%, HAp-Mg 5%, and HAp commercial at different concentrations, and cell survival assays were performed (MTT and AlamarBlue assays).

**Figure 8:**
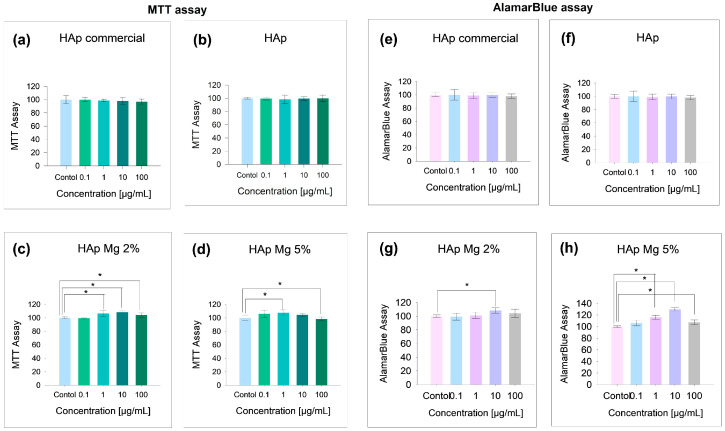
Impact of different hydroxyapatites on fibroblast viability. Results include (**a**–**d**) MTT assay and (**e**–**h**) AlamarBlue assay. Experiments were performed in triplicate; bar graphs show mean values ± SE. * ANOVA test with *p* < 0.05 for significance.

**Figure 9:**
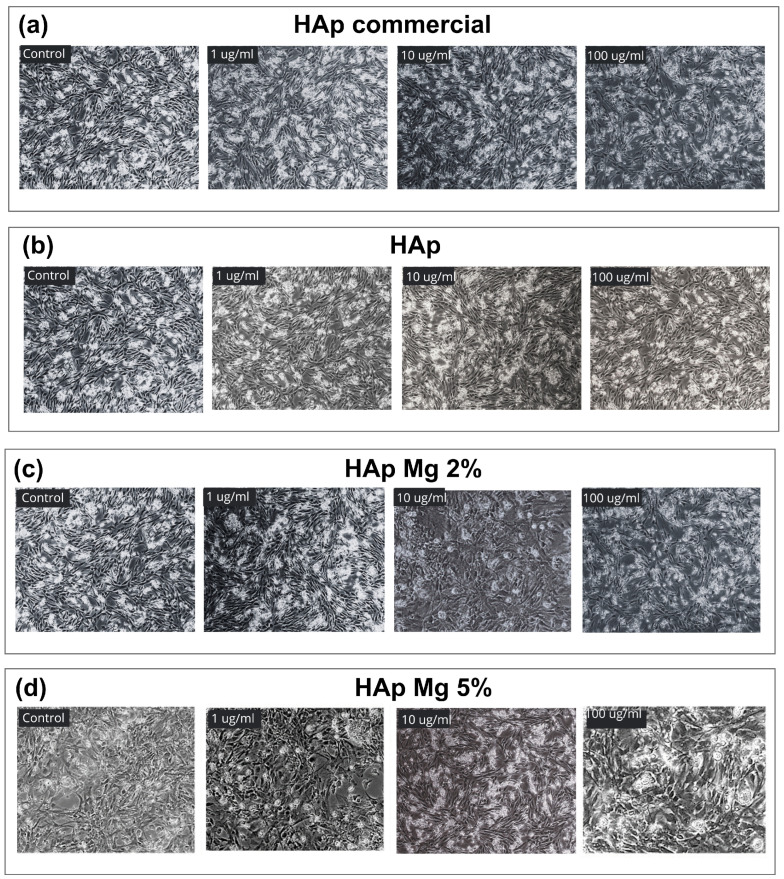
Cellular response of chicken embryo fibroblasts to hydroxyapatite-based treatments. Light microscopy images (10×) taken 24 h post-treatment with (**a**) commercial HAp, (**b**) pure HAp synthesized by MAHM, (**c**) HAp-Mg 2%, (**d**) HAp-Mg 5%, and the control group.

To assess the impact of HAp-Mg on cell viability, primary fibroblast cell cultures were treated with different concentrations of HAp-Mg 2%, HAp-Mg 5%, pure HAp by MAHM, and commercial HAp for 24 h. Each experiment was performed by triplicate. The cell viability and cell proliferations were subsequently assessed using MTT and AlamarBlue assays (Figure 8 and Figure 9).

As shown in Figure 8a, in the MTT assay, no statistically significant differences (*p* > 0.05) in viability were observed between untreated cells (100 ± 3.84%) and cells exposed to HAp-Mg 2% at concentrations of 0.1 µg/mL (99.9 ± 2.75%), 1 µg/mL (98.73 ± 1.94%), 10 µg/mL (97.9 ± 4.92%), and 100 µg/mL (96.8 ± 3.97%). In Figure 8b, in the MTT assay with pure HAp synthesized by MAHM, no statistically significant differences (*p* > 0.05) in viability were observed between untreated cells (100 ± 1.20%) and cells exposed to pure HAp at concentrations of 0.1 µg/mL (99.4 ± 2.09%), 1 µg/mL (98.34 ± 6.57%), 10 µg/mL (99.6 ± 2.53%), and 100 µg/mL (99.9 ± 4.91%). Cell viability of fibroblasts (MTT assay) was not affected even at the highest tested concentration of HAp-Mg 2%. However, a statistically significant increase in cell proliferation was observed at concentrations of 1 µg/mL (106.3 ± 4.55%), 10 µg/mL (108.5 ± 1.78%), and 100 µg/mL (104.2 ± 3.22%), compared to the untreated control (100 ± 1.19%) (Figure 8c).

On the other hand, fibroblasts treated with HAp-Mg 5% showed statistically significant differences (*p* = 0.004) when compared to the control (100 ± 2.03%), particularly at the concentration of 0.1 µg/mL (105.9 ± 5.25%), indicating an increase in cell viability at this concentration. The same trend was observed when comparing the control with HAp-Mg 5% at concentrations of 10 and 100 µg/mL (104.4 ± 2.09% and 98.32 ± 2.322, respectively) (*p* = 0.001) (Figure 8d). However, at the highest concentration (100 µg/mL), a slight decrease in cell viability was observed, possibly due to a mildly cytotoxic effect. Regarding the results of the AlamarBlue assay on cells treated with commercial HAp (Figure 8e) and pure HAp synthesized by MAHM (Figure 8f), no significant differences in cell viability were found compared to the control (*p* > 0.05).

The percentage of live cells did not significantly decrease with either commercial or pure HAp at the different concentrations tested. An interesting finding is that at concentrations of 10 µg/mL of HAp-Mg 2% (107.5 ± 1.26%), cell viability increased above the untreated control group (100 ± 1.97%) (Figure 8g). Similarly, in Figure 8h, a significant increase in fibroblast cell viability was observed at concentrations of 1 µg/mL (106.1 ± 4.79%), 10 µg/mL (116.2 ± 3.57%), and 100 µg/mL (107.6 ± 3.92%) of HAp-Mg 5% compared to the untreated control (100 ± 1.51%) (*p* = 0.021, *p* = 0.001, and *p* = 0.034, respectively).

In addition, the images in Figure 9 reveal no observable reduction in fibroblast viability across all treatments, indicating that neither pure nor Mg-doped HAp compromised cell health compared to the control.

### 2.3. Evaluation of the Antimicrobial Activity of HAp Pure and HAp-Mg

The antimicrobial activity tests by the disk diffusion method of the pure HAp synthesized by MAHM and the HAp-Mg 2% against the different microbial strains tested are summarized in Table 4. The results show the average diameter of the inhibition zones from three independent experiments. No zones of inhibition were observed that reflected the antimicrobial activity of the discs impregnated with different concentrations of commercial HAp (1, 10, and 100 μg/mL); therefore, these data are not included in Table 4.

**Figure 10:**
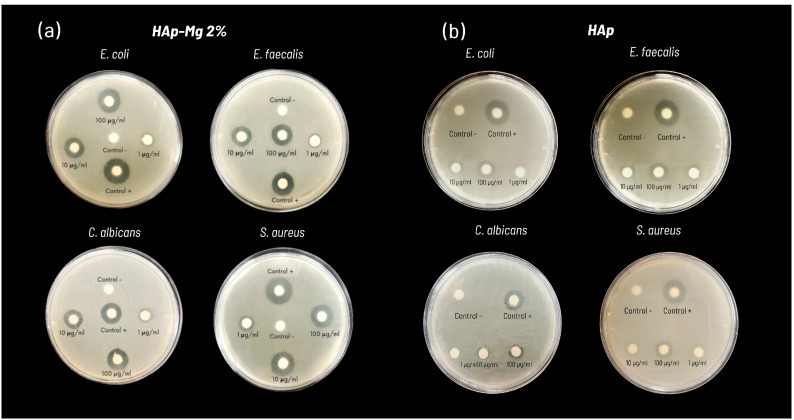
Antimicrobial susceptibility testing results using (**a**) pure HAp and (**b**) HAp-Mg 2% against various microbial strains, demonstrating observed inhibition halos in a representative experiment.

Figure 10 presents the result of a representative experiment of the antimicrobial susceptibility test using the disc diffusion method for each of the strains tested, which include Gram-positive (*Staphylococcus aureus* and *Enterococcus faecalis*), Gram-negative (*Escherichia coli*), and yeast (*Candida albicans*). Concentrations of 1, 10, and 100 μg/mL of HAp were evaluated on each Mueller–Hinton agar plate along with the respective positive and negative controls. Characteristic inhibition halos are observed in suspensions with antimicrobial activity with these being more intense and defined with HAp-Mg compared to pure HAp synthesized by MAHM (Figure 10). The analysis of the inhibition zones indicates that the HAp-Mg 2% nanocomposite powder demonstrates a moderate bactericidal and antifungal effect after 48 or 72 h of incubation at 37 °C at all concentrations evaluated, compared to the control positive (Figure 10a). In contrast, pure HAp shows small zones of inhibition against the strains of bacteria and fungi tested at the concentration of 100 μg/mL (Figure 10b).

**Figure 11:**
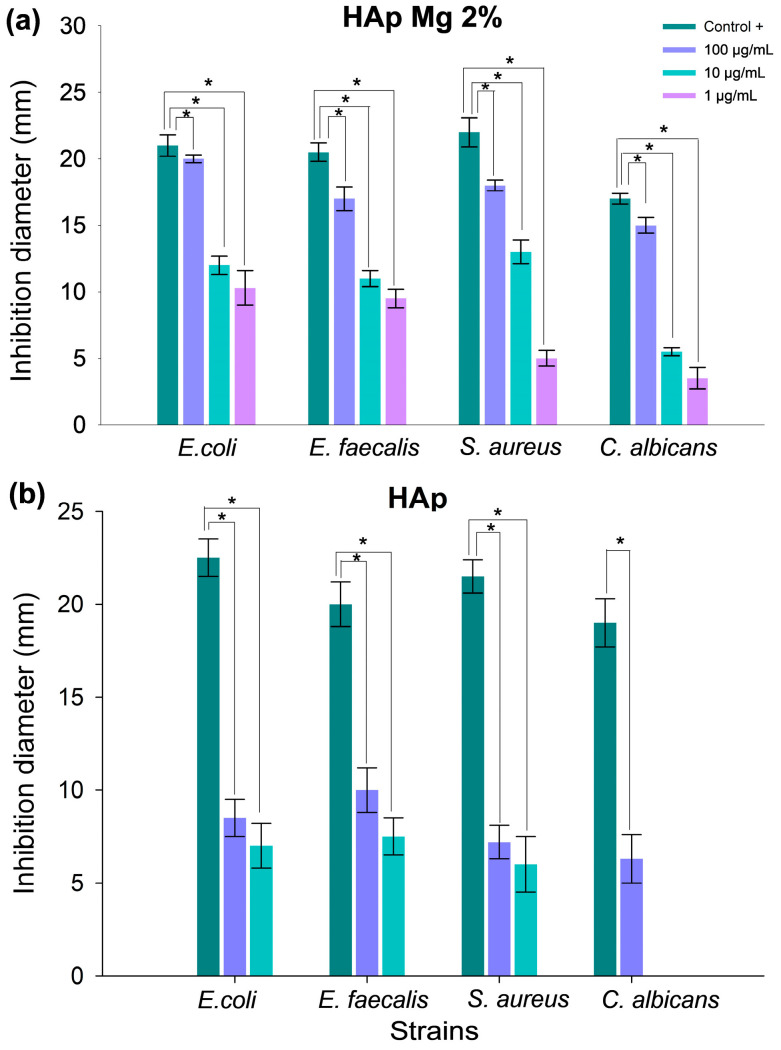
Evaluation of antimicrobial efficacy of nanohydroxyapatite samples against bacterial and fungal strains. Panels depict antimicrobial activity assays using the disk diffusion method with (**a**) HAp-Mg 2% and (**b**) pure HAp synthesized by MAHM. Graphs show average inhibition zone diameters from three independent experiments at different concentrations against bacterial and fungal strains. Results are presented as means with standard error bars. * ANOVA test was employed for inter-group comparisons with significance set at *p* < 0.05.

Figure 11 presents the graph of the average inhibition halo diameters for the tested strains, displaying the results of three independent experiments and comparative analysis with the control group using the ANOVA test. The HAp-Mg 2% nanocomposite exhibits greater antimicrobial activity than pure nanocrystalline HAp synthesized by MAHM (Figure 11). The efficacy of the compound increases with HAp concentration, being particularly significant at a concentration of 100 µg/mL. The following average inhibition halo diameters were observed for HAp-Mg 2% at a concentration of 100 µg/mL: 20.0 ± 0.32 cm against *E. coli*, 17.0 ± 0.95 cm against *E. faecalis*, 18.0 ± 0.41 cm against *S. aureus*, and 15.0 ± 0.61 cm against *C. albicans* (Figure 11a). Statistical analysis comparing the antimicrobial activity of HAp-Mg 2% at concentrations of 0.1, 1, 10, and 100 µg/mL with the control group revealed significant differences among all groups (*p* < 0.05) (Figure 11a).

Regarding pure HAp synthesized by MAHM, it was found that at a concentration of 0.1 µg/mL, there was no antimicrobial activity against any tested strain. Similar to HAp-Mg 2%, maximum antimicrobial effect was observed at a concentration of 100 µg/mL, with the following average inhibition halo diameters: 8.5 ± 1.04 cm against *E. coli*, 10.0 ± 1.25 cm against *E. faecalis*, 7.2 ± 0.98 cm against *S. aureus*, and 6.3 ± 1.38 cm against *C. albicans* (Figure 11b). Significant differences were observed when comparing the average inhibition halo diameters of pure HAp at concentrations of 10 and 100 µg/mL with the control group for all tested strains (*p* < 0.05) (Figure 11b).

**Figure 12:**
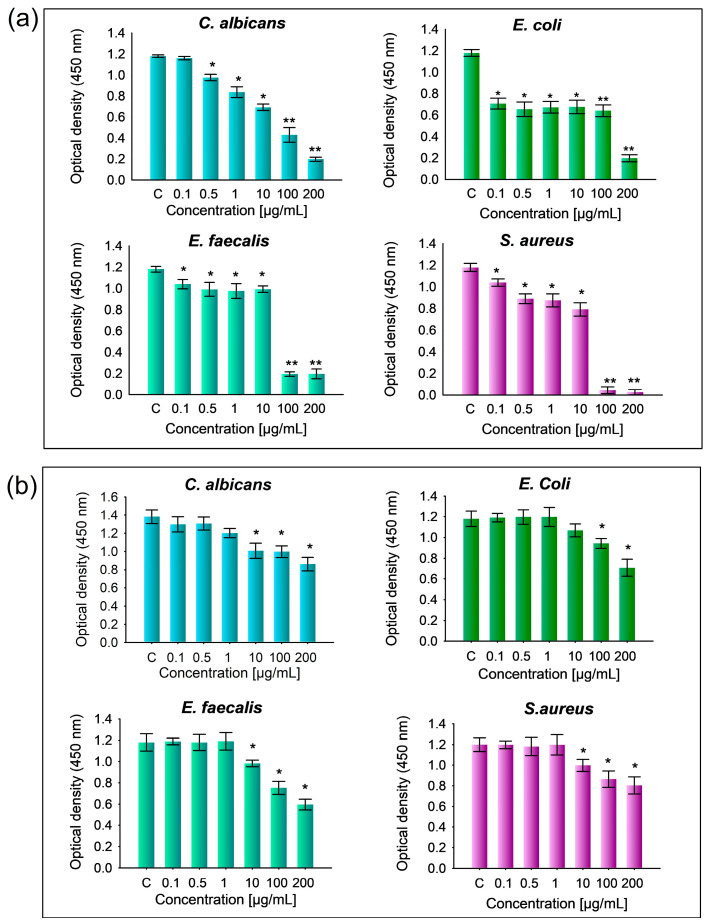
Minimum inhibitory concentration (MIC) assay results for hydroxyapatite (HAp) samples. Error bars represent the variability in MIC values based on different HAp concentrations tested against various microorganisms in comparison to the positive control group. (**a**) HAp-Mg 2% and (**b**) pure HAp synthesized using MAHM. Statistical significance was determined using one-way ANOVA, with *p*-values set at *p* ≤ 0.05 (*) and *p* ≤ 0.01 (**).

Finally, a minimum inhibitory concentration (MIC) assay of HAp-Mg 2% and pure HAp was conducted in triplicate using strains of *E. coli*, *E. faecalis*, *S. aureus*, and *C. albicans*. A comparative analysis was performed with a positive control for each strain. The results are presented in Figure 12, showing the readings obtained in a spectrophotometer at 450 nm. It can be observed that different concentrations of HAp-Mg 2% (0.1, 1, 10, 100, and 200 µg/mL) were evaluated for each microbial strain to determine the minimum concentration capable of inhibiting bacterial or fungal growth. The error bars provide an indication of variability among experiment repetitions.

The results obtained for HAp-Mg 2% were as follows: for *C. albicans*, the control showed an absorbance of 1.18 ± 0.11. As the concentration of HAp-Mg 2% increased, the absorbance values decreased progressively: 0.5 µg/mL (1.18 ± 0.016), 1 µg/mL (0.836 ± 0.051), 10 µg/mL (0.69 ± 0.030), 100 µg/mL (0.43 ± 0.069), and 200 µg/mL (0.19 ± 0.014). For *E. coli*, the control had an absorbance of 1.21 ± 0.031 (Figure 12a).

With HAp-Mg, the absorbance values were as follows: 0.1 µg/mL (0.70 ± 0.050), 0.5 µg/mL (0.65 ± 0.076), 1 µg/mL (0.67 ± 0.053), 10 µg/mL (0.67 ± 0.062), 100 µg/mL (0.64 ± 0.053), and 200 µg/mL (0.20 ± 0.032). *E. faecalis* exhibited a control absorbance of 1.18 ± 0.028. With increasing concentrations of HAp-Mg 2%, absorbance values were 0.1 µg/mL (1.04 ± 0.043), 0.5 µg/mL (0.99 ± 0.069), 1 µg/mL (0.97 ± 0.065), 10 µg/mL (0.99 ± 0.030), 100 µg/mL (0.19 ± 0.021), and 200 µg/mL (0.19 ± 0.045). *S. aureus* showed a control absorbance of 1.20 ± 0.038 (Figure 12a).

With HAp-Mg 2% treatment, absorbance values were as follows: 0.1 µg/mL (1.04 ± 0.033), 0.5 µg/mL (0.89 ± 0.045), 1 µg/mL (0.87 ± 0.059), 10 µg/mL (0.79 ± 0.030), 100 µg/mL (0.045 ± 0.031), and 200 µg/mL (0.02 ± 0.025). These results indicate a concentration-dependent decrease in absorbance with HAp-Mg treatment across all tested strains. Significant differences were observed compared to the control in several concentrations, underscoring the effectiveness of HAp-Mg 2% in inhibiting microbial growth (Figure 12a).

The results obtained for pure HAp synthesized by MAHM were the following: for *C. albicans*, the control had an absorbance of 1.38 ± 0.07. As the concentration of HAp increased, the absorbance values decreased progressively: 0.5 µg/mL (1.30 ± 0.07), 1 µg/mL (1.20 ± 0.032), 10 µg/mL (1.08 ± 0.052), 100 µg/mL (0.99 ± 0.042), and 200 µg/mL (0.86 ± 0.046). For *E. coli*, the control showed an absorbance of 1.18 ± 0.07 (Figure 12b).

With increasing concentrations of pure HAp, the absorbance values were as follows: 0.1 µg/mL (1.19 ± 0.013), 0.5 µg/mL (1.19 ± 0.029), 1 µg/mL (1.89 ± 0.092), 10 µg/mL (1.06 ± 0.075), 100 µg/mL (0.94 ± 0.047), and 200 µg/mL (0.70 ± 0.08). *E. faecalis* exhibited a control absorbance of 1.19 ± 0.08. As the concentration of HAp increased, absorbance values were as follows: 0.1 µg/mL (1.19 ± 0.033), 0.5 µg/mL (0.18 ± 0.042), 1 µg/mL (1.19 ± 0.081), 10 µg/mL (0.98 ± 0.015), 100 µg/mL (0.75 ± 0.010), and 200 µg/mL (0.59 ± 0.050), and *S. aureus* showed a control absorbance of 1.19 ± 0.046. With HAp treatment, absorbance values were as follows: 0.1 µg/mL (1.19 ± 0.073), 0.5 µg/mL (1.18 ± 0.065), 1 µg/mL (1.19 ± 0.097), 10 µg/mL (0.99 ± 0.058), 100 µg/mL (0.86 ± 0.079), and 200 µg/mL (0.80 ± 0.082) (Figure 12b). These results indicate varying degrees of absorbance change with increasing concentrations of pure HAp across different microbial strains. Significant differences were observed in absorbance values compared to the control, indicating the effectiveness of pure HAp at different concentrations influencing microbial activity (Figure 12b).

Overall, the results demonstrate that as the concentration of HAp-Mg 2% increases, there is a decrease in absorbance at 450 nm, indicating greater inhibition of microbial growth (Figure 12a). The strongest inhibitory effect on microbial growth is observed at a concentration of 200 µg/mL of HAp-Mg 2% across all analyzed strains. Additionally, significant differences are observed between the evaluated strains and the positive control (*p* < 0.05) (Figure 12a), suggesting the efficacy of HAp-Mg 2% as an antimicrobial agent against the test strains. In contrast, with pure HAp, a lesser inhibition effect on microbial growth is noted compared to HAp-Mg 2%, becoming evident at concentrations as low as 10 µg/mL without achieving complete inhibition of bacterial or fungal growth even at the highest concentration tested (Figure 12b). Significant differences in the optical density of microbial cultures treated with pure HAp compared to the untreated control are observed from a concentration of 10 µg/mL onwards (*p* < 0.05) (Figure 12b).

## 3. Discussion

Our results demonstrate that the pure HAp and HAp-Mg nanofibers obtained via the microwave-assisted hydrothermal method exhibit a specific structural organization and a high degree of purity and crystallinity. These characteristics could potentially enhance mechanical resistance and biocompatibility in in vivo studies. The inclusion of glutamic acid in the pure HAp and HAp-Mg synthesis reaction facilitated crystalline growth, thereby enhancing its properties, as suggested in our previous studies [18,19]. SEM morphological analysis findings indicate that pure HAp, HAp-Mg 2%, and HAp-Mg 5% nanofibers display a defined hexagonal morphology, suggesting a uniform growth pattern during synthesis. This hexagonal shape is indicative of the well-defined crystallographic structure of HAp, consistent with our previous findings [19,20]. Moreover, we observed that the incorporation of 2% or 5% magnesium ions minimally alters the overall morphology of the HAp. The nanofibers present smooth surfaces and well-defined ends with no irregularities or defects, indicating homogeneous material deposition and controlled growth conditions. Quantitative analysis of the SEM images revealed an average diameter of approximately 402 nm for the HAp-Mg nanofibers, with lengths ranging from 20 to 30 μm. This uniformity confirms the reproducibility and precision of the MAHM in fabricating nanofibers.

The interconnected network of HAp-Mg 2% and HAp-Mg 5% nanofibers observed in the SEM images suggests the formation of a hierarchically organized three-dimensional structure beneficial for applications requiring enhanced mechanical strength and surface area. Additionally, these nanofibers exhibit a spherical distribution, with an approximate size of 25 µm. This spherical morphology implies the presence of agglomerates or nanofiber bundles, possibly arising from electrostatic interactions or physical entanglements during synthesis. Similar phenomena have been reported by Zhou et al. (2012), who observed the formation of magnesium phosphate nanospheres promoting osteoblast cell proliferation [21]. The presence of these nanospheres in our study may stem from interactions between magnesium ions and phosphate groups in the precursor solution.

The formation of HAp-Mg 2% and HAp-Mg 5% involves a complex process initiated by the adsorption of magnesium ions (Mg^2+^) to phosphate groups, resulting in magnesium–phosphate complexes in solution. These complexes may serve as growth nuclei for HAp-Mg nanosphere formation under specific pH, temperature, and precursor concentration conditions. During nucleation and growth, magnesium ions can influence the structure and morphology of HAp crystals, contributing to nanosphere formation instead of other crystalline forms [21]. Additionally, electrostatic interactions and Van der Waals forces between nanostructures may promote self-assembly and agglomeration of HAp-Mg cores, leading to nanosphere or nanocrystal aggregate formation, as observed in our study.

In contrast, in the SEM micrographs of the commercial HAp nanoparticles, we observed an amorphous and heterogeneous form that, in turn, formed irregular conglomerates, being less crystalline and organized than HAp-Mg 2%. Furthermore, we found that as the Mg^2+^ concentration increased (5%) in the HAp-Mg 5%, its crystalline quality decreased, indicating an influence of the dopant element abundance on the structural configuration of HAp.

Additionally, the uniform distribution of the constituent elements of the pure HAp and HAp-Mg nanofibers synthesized by MAHM was demonstrated by EDS analysis. Using the obtained data, the elemental composition of the samples analyzed was calculated. The results revealed that the Ca/P ratio of pure HAp and HAp-Mg 2% was more favorable compared to the commercial HAp and HAp-Mg 5% samples, showing a value close to 1.66 and 1.67, respectively, consistent with the typical stoichiometry of HAp. This finding is relevant since an adequate Ca/P ratio is essential to guarantee the stability and optimal integration of HAp with the surrounding tissue. A significant deviation from this relationship can negatively impact the ability of HAp to bind to adjacent bone tissue, affecting its biodegradability and osseointegration capacity.

It is essential to highlight that in the biomedical context, the synthetic HAp used is intended to have a Ca/P ratio similar to that of natural bone, typically in the range of 1.67 to 1.70. This correspondence is crucial as it influences the chemical stability, biocompatibility, and interaction capacity of HAp with biological tissues [1].

Elemental analysis found that the magnesium content in the nanofibers was well below the threshold level that could induce cytotoxicity in vitro and in vivo, indicating the good biocompatibility of the material. EDS analysis also revealed a uniform distribution of magnesium ions in the HAp network, suggesting that the doping process was successful in producing homogeneous HAp nanofibers.

On the other hand, the X-ray diffraction analysis of the pure HAp, HAp-Mg 2%, and HAp-Mg 5% revealed a series of well-defined Bragg reflections in the obtained diffractograms. These reflections correspond to the characteristic crystalline planes of the HAp crystal structure, as expected, and agree with the data from the Powder Diffraction File of ICDD #09-432.

The Bragg reflections observed in the diffractograms are related to the Miller indices of the crystalline planes of HAp, such as (211) for the main peak at a 2θ angle of 31.82 for HAp-Mg 2%, HAp-Mg 5%, and commercial HAp; (300), which corresponds to a 2θ angle of 33.79° and the main peak at a 2θ angle, for pure HAp synthesized by MAHM; (002), which corresponds to a 2θ angle of 25.91°; among other characteristic signals. These reflections represent the typical X-ray diffraction pattern through the atomic planes of the HAp crystal lattice, confirming the presence of this phase in the analyzed samples. It is important to mention that background noise was also observed in the diffractograms, especially in the case of commercial HAp. This noise may be due to the presence of impurities or the presence of amorphous constituents in the samples, although to a lesser extent. However, the intensity of this background noise is low compared to the main diffraction signals of HAp, suggesting that the main phase present in the samples is HAp-Mg (2 and 5%), pure HAp, and commercial HAp.

Regarding magnesium doping, it was observed that as the magnesium concentration increases in the synthesis of HAp, a decrease in the intensity of Bragg reflections occurs. This effect was observed in the intensity of the (300) reflection, and it suggests the possible reduction in the preferential crystalline orientation of HAp due to the presence of magnesium ions in the crystal lattice. The Bragg (211) reflection was the most intense in all the diffractograms of the samples analyzed (commercial HAp, HAp-Mg 2%, and HAp-Mg 5%) and occurred at a diffraction angle of approximately 31.8° at the 2θ angle in the diffractograms, in accordance with the Miller indices of the crystalline planes of HAp.

In the scanning electron microscopy (SEM) results, it was observed that pure HAp and HAp-Mg 2% present a more crystalline and organized morphology compared to commercial HAp and HAp-Mg 5%. Additionally, the diffractogram of the pure HAp and HAp-Mg 2% exhibited well-defined, narrow, and intense signals, with little background noise, corresponding to the Bragg reflections of the crystalline planes characteristic of this compound. This indicates a well-developed crystal structure and the high quality and purity of the samples. Furthermore, this observation supports the hypothesis that magnesium concentration influences the structure and crystallinity of HAp, as reported by other authors [22,23,24].

Overall, the concentration increment of the doping ions in the HAp synthesis mixture reduces the average crystallite size and increases the potential content of amorphous materials in the sample. The last produced a lower degree of crystallinity of HAp. These findings highlight the importance of considering the effect of magnesium doping on the structure and properties of HAp, which has significant implications for its applicability in bone regeneration [24]. However, additional studies are needed to fully understand the mechanisms behind these effects and optimize magnesium doping to improve HAp properties.

In addition to this information, the characterization of HAp samples by the Fourier transform infrared spectroscopy (FTIR) technique revealed the presence of several relevant functional groups. In the FTIR spectrum, characteristic functional groups of HAp and possible compounds resulting from magnesium doping were identified. Bands corresponding to phosphate groups were observed, typically around 900–1200 cm^−1^, which are characteristic of the HAp structure. In addition to these functional groups, the doping introduced new characteristics in the FTIR spectrum, such as the presence of magnesium evidenced by the peaks at 536 cm^−1^, corresponding to the vibrations of the Mg–O bonds. This peak exhibited an intensity of 40.748, indicating substantial incorporation of magnesium ions into the HAp crystal lattice. However, all the characteristic vibration bands of HAp were evident, with some changes resulting from doping. These variations are similar to what were reported in other characterization studies of magnesium-doped HAp [15,16,25,26].

However, our study explored the potential mechanism of action of magnesium in improving the biological properties of nanohydroxyapatite, particularly its effect on cell viability and proliferation observed in fibroblasts treated with HAp-Mg at different concentrations, compared with commercial HAp and pure HAp. For this, the MTT and AlamarBlue assays were performed, both commonly used to measure cell viability and proliferation. In the MTT assay, viable cells metabolized the MTT reagent into a colored formazan product quantifiable by spectrophotometry. This assay measures cell viability by evaluating the redox potential of the tested cells [27]. The results obtained from the MTT assay in fibroblasts cultured under the experimental conditions showed that, although the higher concentrations of HAp-Mg 2% and HAp-Mg 5%, as well as commercial HAp, tended to slightly reduce cell viability compared to the control, these changes did not reach statistical significance. However, it is worth highlighting that a significant increase in cell viability in samples treated with HAp-Mg 2% at concentrations of 1, 10, and 100 µg/mL for 24 h. Specifically, the concentration of 10 µg/mL of HAp-Mg 2% and HAp-Mg 5% showed the greatest increase in cell viability, being statistically significant compared to the untreated control group. The results of the MTT assay indicated a minimal cytotoxic effect of HAp-Mg 5% and commercial HAp at concentrations of 100 µg/mL (less than 5%), while HAp-Mg 2% did not present cytotoxic effects at the concentrations evaluated. Therefore, this assay proved to be a reliable and sensitive method to assess the viability of fibroblasts treated with different concentrations of HAp.

AlamarBlue is a sensitive indicator of oxidation–reduction mediated by mitochondrial enzymes and fluoresces, and it changes color upon reduction by living cells [28]. The AlamarBlue assay has been reported to have superior sensitivity to the MTT assay for assessing drug cytotoxicity in cultured cells [29]. In our study, we observed similar results between the MTT assay and AlamarBlue, suggesting minimal and non-significant cytotoxicity compared to control in fibroblast samples treated with 100 µg/mL concentrations of pure HAp, commercial HAp, HAp-Mg 2%, and HAp-Mg 5%. Furthermore, the results of the AlamarBlue assay showed a statistically significant increase in fibroblast proliferation induced by HAp-Mg 2%, especially at concentrations of 10 µg/mL, and by HAp-Mg 5% at concentrations of 1 and 10 µg/mL, probably due to the presence of the doping element. These effects were not observed in commercial HAp or pure HAp synthesized by MAHM, suggesting that our HAp-Mg has a greater impact on inducing cell proliferation than pure commercial HAp.

The role of magnesium in cell proliferation is of interest due to its importance for various cellular and biological functions in the human body. Magnesium is the second most abundant intracellular cation, with a concentration of around 1000 mmol or 24.0 g, the majority of which is stored in bones [22]. This ion participates in the regulation of ion channels, the stabilization and duplication of DNA, as an enzymatic cofactor and catalyst of metabolic reactions, and in the stimulation of cell growth and proliferation [24,29]. Additionally, magnesium participates in the regulation of the proliferation of eukaryotic cells. In response to mitogenic factors, intracellular Mg increases in the G1 and S phases of the cell cycle, and this event correlates with the enhancement of protein synthesis and the initiation of DNA synthesis. Later in the cell cycle, Mg^2+^ contributes to mitotic spindle formation and cytokinesis [30]. Consequently, Mg^2+^ regulates the cell cycle and stimulates cell proliferation.

For this reason, magnesium doping for some ceramics has been studied in depth in recent years [22]. The concentration of HAp-Mg has been observed to affect its properties, such as crystallinity and solubility. An increase in the concentration of Mg^2+^ leads to a decrease in the crystallinity of HAp; however, there is an increase in its solubility attributed to the substitution of calcium ions for magnesium ions in the crystal lattice of HAp [11,24].

Furthermore, HAp-Mg has been shown to have beneficial effects on osteoblast proliferation and differentiation, as well as on osseointegration and osteoconductivity in biomedical implants, leading to better acceptance and functionality of implants coated with this biomaterial. Additional studies have supported these findings. For example, Bodhak et al. (2012) evaluated the effects of dopant ions, Mg and Sr, on the biological properties and structural stability of HAp in human osteoblast and osteoclast cell cultures. They observed an improvement in the density of live cells, measured through the MTT assay, after 7 days of treatment, with results consistent with those obtained in our study [31]. Furthermore, they highlighted that the stability of HAp increased with the presence of Mg and Sr ions, suggesting a positive influence of magnesium ions on the mineral metabolism that occurs during bone remodeling. Likewise, it was found that Mg^2+^ promotes the proliferation of preosteoblastic cells and osteoblasts, as well as the apoptosis of osteoclasts, which favors rapid and effective tissue regeneration. This aligns with previous studies that have demonstrated the regulatory role of Mg^2+^ in osteoclast-mediated bone remodeling [32,33]. For example, Rude et al. (2009) conducted experiments in magnesium-deficient rodents, showing an increase in bone loss, possibly due to the increase in osteoclast-mediated resorption because of magnesium deficiency [33]. This suggests that the adequate presence of magnesium is crucial for maintaining bone homeostasis.

Other studies have shown that Mg^2+^ deficiency is associated with an increase in the number of osteoclasts derived from bone marrow precursors, although their bone remodeling activity is decreased under such conditions. This is attributed to the activation of transcription factors and key regulatory proteins of osteoclastogenesis, as well as the elevated expression of proinflammatory cytokines, such as TNF-α and IL-1β, which contribute to the decrease in osteoclast activity under conditions of Mg^2+^ deficiency [32]. Together, these results support the notion that HAp-Mg has the potential to improve the biomimetic and bioactive properties of materials used in biomedical applications, especially bone regeneration.

However, further research is required to fully understand the effects of HAp-Mg and its impact on biological response in vitro and in vivo environments as well as to optimize doping conditions to obtain the desired benefits without compromising the structural and functional integrity of the material.

On another note, the study of the antimicrobial activity of nanomaterials is essential in the search for new strategies to combat bacterial and fungal infections, especially in the context of growing resistance to antibiotics. In our research, we evaluated the antimicrobial activity of HAp samples, focusing on their effect on bacterial and fungal strains relevant to periprosthetic infections.

The results obtained revealed notable antibacterial activity of HAp-Mg nanoparticles, especially against strains of *Staphylococcus aureus* and *Escherichia coli*. This effect was dependent on the concentration of HAp, being more evident at the highest concentrations tested. These findings are consistent with previous studies that have also reported the antimicrobial efficacy of HAp-Mg against a wide variety of pathogens [15,16].

The mechanism underlying the antimicrobial activity of HAp-Mg has garnered considerable attention in the scientific literature. The release of Mg^2+^ ions from the HAp network has been shown to play a crucial role in this process. These ions can alter the structural integrity of bacterial cell walls and membranes, ultimately inducing cell death [34]. Furthermore, the ability of magnesium to induce oxidative stress in bacteria through the generation of reactive oxygen species (ROS) has been well-documented [15,16]. Magnesium can alter the pH of the surrounding environment and promote the generation of reactive oxygen species (ROS) within the bacterial cell [35]. These ROS, like oxygen-free radicals, are highly reactive and can cause oxidative damage to cellular macromolecules, including lipids, proteins, and DNA. This damage can affect cell membrane integrity, protein structure, and genetic material stability, ultimately leading to bacterial cell death [36].

Moreover, HAp-Mg and HAp pure synthesized by MAHM not only exhibited antimicrobial activity but also displayed significant antifungal properties. The antimicrobial effect was greater in HAp-Mg 2% than in pure HAp synthesized by MAHM at the highest concentrations tested. In contrast, commercial HAp did not show any antimicrobial effect.

Recent research has revealed that the presence of Mg^2+^ in the HAp-Mg structure has a disruptive effect on the synthesis of ergosterol, an essential component of the cell membrane in fungi [34]. This interaction leads to a significant alteration of membrane integrity and cell permeability in fungi, resulting in inhibition of the growth and viability of these microorganisms [36]. Ergosterol plays a crucial role in the structure and function of the fungal cell membrane, acting as an essential lipid component that contributes to its integrity and permeability. However, the presence of Mg^2+^ in the HAp-Mg matrix interferes with normal ergosterol biosynthesis, which alters the lipid composition of the membrane and compromises its protective function [37]. As a result, decreased ergosterol levels in the cell membrane compromise its structural integrity and its ability to maintain cellular homeostasis, leading to disruption of the growth and survival of yeasts such as *C. albicans* [15,38]. This mechanism of action gives HAp-Mg significant potential as an antifungal agent in biomedical applications.

## 4. Materials and Methods

### 4.1. Synthesis of HAp-Mg Using Microwave-Assisted Hydrothermal Method

HAp-Mg nanofibers were produced using the microwave-assisted hydrothermal method (MAHM), with calcium nitrate (CaNO_3_), potassium phosphate dibasic (K_2_HPO_4_), and potassium hydroxide (KOH) as precursor substances and glutamic acid to aid in crystal growth. The synthesis process involved several steps: First, a solution of glutamic acid [C_5_H_9_NO_4_-H_2_O] (J.T. Baker FW 147.13; Phillipsburg, NJ, USA) and calcium nitrate tetra hydrate [Ca (NO_3_)_2_-4H_2_O] (Golden Bell FW 236.16; Bell, CA, USA) was prepared in 200 mL of deionized water. The solution was heated and stirred magnetically for approximately two hours until the solutes were completely dissolved. Additionally, monobasic potassium phosphate [KH_2_PO_4_] (Mallinckrodt Chemicals FW 136.09; Chesterfield, UK) and potassium hydroxide [KOH] (Sigma-Aldrich 221473; St. Louis, MO, USA) were dissolved in 200 mL of deionized water using vigorous agitation. Once both solutions were prepared, they were mixed to create the reacting mixture, which was continuously agitated. So far, it is possible to synthesize pure HAp nanofibers with this formulation.

Afterward, drop by drop of 2% or 5% by weight solution of magnesium nitrate Mg (NO_3_)_2_ (MY.6247-100; Meyer Laboratory Inc., Blue Springs, MO, USA) was added in relation to the calcium content in CaNO3, and then this mixture was mechanically agitated for additional 10 min. The main difficulty to overcome was the good dissolution of all components of the mixture, but this was solved by using mechanical agitation and heating the solution up to 60 °C.

The reacting mixture was then transferred to quartz tubes and placed inside a Shyntos 300 (Anton Paar, Ashland, VA, USA) microwave oven. The synthesis reaction was carried out at 170 °C in a reaction time of 45 min, with a heating time of 10 min (since 25 °C) and a cooling time of 10 min (17 °C/minute). This cooling rate was necessary to avoid the fragmentation of fibers when cooling took place naturally.

A commercial HAp powder with a particle size of <200 nm (677418, Sigma-Aldrich, St. Louis, MO, USA) was acquired for conducting a comparative structural and characterization analysis.

### 4.2. Characterization of Nanomaterials

#### 4.2.1. X-Ray Diffraction

The HAp-Mg nanofibers were analyzed using the X-ray diffraction by powders technique to identify the crystalline phases within the sample and to determine any possible preferential crystalline orientation. A Bruker D8 Advance Diffractometer (Bruker, Karlsruhe, Germany) was utilized to perform the crystal structure analyses. Cu Kα radiation with a wavelength of 0.15406 nm was produced using an accelerating voltage of 20 kV and a current of 20 mA. The diffraction experiments were carried out from 10° to 80° on a 2θ scale with an angular step of 0.05° and one second dwell time at each angular step. No milling process was performed on any sample. Lattice parameters were determined for pure and Mg-doped HAp using Rietveld refinement to evaluate the entry of the magnesium ions into the HAp crystal structure. All diffractograms were processed using open-source software Profex (version 5.3.1, available at https://www.profex-xrd.org/ (accessed on 2 May 2024)).

#### 4.2.2. Scanning Electron Microscopy

The morphology and topology of the HAp nanofibers were observed using two scanning electron microscopes: a JEOL JSM-6060 LV (Jeol Ltd., Akishima, Tokyo, Japan) and a Field Emission Scanning Electron Microscope (FE-SEM) model SU8200 (Hitachi High-Technologies, Tokyo, Japan). Accelerating voltages of 10 kV and 20 kV were applied to perform the observations, and secondary electrons were used to form all images. Dry samples were collected directly from the synthesis process, placed on a sample holder, and glued with carbon paint.

#### 4.2.3. Transmission Electron Microscopy

Morphology and crystalline details of nanofibers were observed by Transmission Electron Microscopy (TEM). These observations were carried out using a JEOL JEM 2010HT (Jeol Ltd., Akishima, Tokyo, Japan) using an accelerating voltage of 200 kV. Samples were previously dispersed in ethanol, and one drop was placed on a carbon-coated Cu grid with a 3 mm diameter.

#### 4.2.4. Energy-Dispersive X-Ray Spectroscopy Analysis

Elemental chemical microanalysis was conducted using energy-dispersive spectroscopy (EDS) with a Quantax EDS system (Bruker Nano GmbH, Berlin, Germany). Specimens of pure and Mg-doped HAp were coated with gold/palladium for 1 min using a magnetron sputter coater from Emitech Inc. The gold-coated specimens were observed using an FE-SEM model SU8200 (Hitachi High-Technologies, Tokyo, Japan). The specimens used in the SEM study were 7 mm in diameter and 2.2 mm in thickness.

The SEM-EDX spectra were performed using a JEOL JSM-6060 LV (Jeol Ltd., Akishima, Tokyo, Japan) equipped with an X-MaxN 80 mm silicon drift detector (Oxford Instruments, Oxford, UK). For SEM and EDX analyses, an accelerating voltage of 10 kV was used.

#### 4.2.5. FTIR-ATR

The ATR-FTIR spectra of HAp-Mg were acquired on a Nicolet Summit PRO FTIR Spectrometer (Thermo Fisher Scientific, Waltham, MA, USA) with a Smart Orbit single-diamond ATR element at a resolution of 8 cm^−1^ and 32 co-added scans in the wavenumber range of 4000–400 cm^−1^.

### 4.3. Biocompatibility, Cell Viability, and Cytotoxicity In Vitro Experiments

#### 4.3.1. Experimental Animals

Fertilized eggs used in this study (*Gallus domesticus*, *White Leghorn*) were obtained from Pilgrim’s Pride (Querétaro, México). The eggs were incubated at 39 °C in a humidified air chamber (IAMEX, Mexico City, México) and rotated one-quarter of a revolution every 50 min during incubation. All experimental procedures were conducted according to national (NOM-062-ZOO-1999) and international guidelines (The Guide for Care and Use of Laboratory Animals, U.S. National Research Council).

#### 4.3.2. Fibroblast Cell Cultures and Treatments with HAp-Mg

Chick embryos at 12 days of embryogenesis were anesthetized in ovo by cooling them on ice for 5 min and then euthanized by decapitation. Next, the skin from the head was micro-dissected in a cold, calcium-/magnesium-free Hank’s balanced buffer using a microsurgical blade under a stereoscopic microscope. The tissue was then digested in a 0.25% trypsin-EDTA solution (Sigma-Aldrich, St. Louis, MO, USA) at 37 °C for 10 min, followed by mechanical dissociation with a glass pipette. Cells were counted using the trypan blue exclusion method in a Neubauer chamber, and (1 × 105) cells were plated in 96-well plates (Costar, Corning, NY, USA).

The fibroblasts were then incubated in DMEM (Gibco, Grand Island, NY, USA) containing 10% fetal bovine serum (Gibco, Grand Island, NY, USA) and 1% penicillin-streptomycin (Gibco, Grand Island, NY, USA) for 3 days at 39 °C in a humidified incubator (Forma Steri-Cycle i160, Thermo Scientific, Waltham, MA, USA) under an atmosphere of 95% air/5% CO_2_. For treatments, HAp-Mg 2% or HAp-Mg 5% was dissolved in dimethyl sulfoxide (DMSO) 0.25% (Thermo Scientific™ 20688, Waltham, MA, USA) and added to the fibroblast at various concentrations (0.1, 1, 10, or 100 µg/mL) for 24 h. A negative control group of cells was incubated without HAp-Mg. The fibroblasts were visualized using an Olympus IX53 inverted microscope (Olympus Corp., Tokyo, Japan).

#### 4.3.3. Cell Survival and Cytotoxicity Assay

Cell survival was assessed indirectly using two assays: the MTT (3-[4,5-dimethylthiazol-2-yl]-2,5-diphenyltetrazolium bromide) assay and the AlamarBlue™ assay (Invitrogen, Thermo Fisher Scientific, Waltham, MA, USA). For the MTT assay, following HAp-Mg treatments, the culture medium was replaced with DMEM medium without phenol red (Gibco, Grand Island, NY, USA), and the MTT reagent (Thermo Scientific, Waltham, MA, USA) was added to each well at a final concentration of 0.5 mg/mL. The plates were then incubated for 4 h at 37 °C in a humidified incubator (Forma Steri-Cycle i160, Thermo Scientific, Waltham, MA, USA). The resulting formazan crystals were dissolved in 0.1 mL of solubilization solution (DMSO), and the products were finally spectrophotometrically quantified at 570 nm using a Multiskan™ FC Microplate Photometer (Thermo Fisher Scientific, Waltham, MA, USA).

For the AlamarBlue assay, 200 µL of DMEM medium without phenol red supplemented with 10% AlamarBlue was added to each well, and the plates were incubated for 4 h in a humidified atmosphere at 37 °C and 5% CO_2_. The colorimetric detection was monitored by measuring the absorbance at 570 nm using a Multiskan™ FC Microplate Photometer (Thermo Fisher Scientific, Waltham, MA, USA).

#### 4.3.4. Antimicrobial Activity Evaluation of HAp-Mg

The antimicrobial activity of the samples of HAP-Mg dissolved in DMSO in a concentration of 100 μg/mL was tested against four bacterial strains: *Escherichia coli* (ATCC^®^ 25922), *Staphylococcus aureus* (ATCC^®^ 25923), *Enterococcus faecalis* (ATCC^®^ 29212), as well as a yeast strain, *Candida albicans* (ATCC^®^ 24433). Inoculum of all microorganisms was prepared from fresh overnight broth cultures (Tripton soy broth with 0.6% yeast extract—Torlak, Belgrade), which were incubated at 37 °C. The resulting broth cultures were used for the tests. The agar diffusion test was performed using Mueller–Hinton solid agar (Institute for Immunology and Virology, Torlak, Belgrade).

The disc diffusion method was carried out by pouring the Mueller–Hinton agar into Petri dishes to form 4 mm thick layers. With the help of a densitometer (Fisherbrand™ Thermo Fisher Scientific, MA, USA), turbidity was measured with absorbances of 0.08–0.1 for bacteria, equivalent to 0.5 of the McFarland standards, obtaining a resulting microbial suspension of 1–2 × 10 (UFC/mL). After adjusting the turbidity of the inoculum suspension, the entire surface of a Mueller–Hinton agar plate was inoculated with a sterile swab (Deltalab, S.L., Spain). Subsequently, in sterile conditions, a disc with HAp-Mg solution, a disc with positive control, and a disc with negative control were arranged on the agar surface. Oxacillin (1 μg) was used as a positive control for *S. aureus*, Ciprofloxacin (5 μg) was used for *E. faecalis* and *E. coli*, and Miconazole (20 mg/mL) was used for *C. albicans*. Also, DMSO was used as a negative control.

The plates were incubated for 24 and 48 h at 37 °C ± 0.5 °C (bacteria) or 48 and 72 h at 25 °C ± 0.5 °C (yeast) in an incubator (EcoshelI, TX, USA). Once the incubation time finished, the diameters of the inhibition halos were measured with the help of a ruler, and the measurements were recorded in millimeters. The tests were performed three times, and the results represented their average values ± standard error. The results were graphed in SigmaPlot Version 15.0 software (SPSS Inc., Chicago, IL, USA).

The minimum inhibitory concentration was performed using a quantitative antimicrobial assay using a serial microdilution method in 96-well plates with Nutrient Broth No.1, Standard Liquid Medium (Merck Millipore, Merck Group, Darmstadt, Germany). Initially, 1000 μL of microbial suspension of each microorganism, with a 106 CFU/mL density, was seeded in each well. The cells were incubated at 37 °C for 24 h in the presence of 0.1, 0.5, 1, 10, 100, and 200 μg/mL of HAp-Mg/DMSO suspension. A microbial suspension without treatment was used as a negative control. The quantification of the antimicrobial activity of the HAp-Mg suspensions against the microorganisms was performed by reading the absorbance of the microbial suspensions at 450 nm in a Multiskan™ FC Microplate Photometer (Thermo Scientific, Waltham, MA, USA). The experiments were performed in triplicate, and the results were presented as means ± SD.

#### 4.3.5. Statistical Analysis

All in vitro experiments were performed in triplicate. The results of the experiments were presented as mean values ± standard error (SE). The results were analyzed using SigmaPlot Version 15.0 software (SPSS Inc., Chicago, IL, USA). The student t-test and ANOVA were used to compare the groups for the inferential data analysis. A *p*-value <0.5 was considered significant.

## 5. Conclusions

In conclusion, the HAp-Mg exhibits promising potential for bone regeneration evaluation due to several significant benefits over undoped commercial HAp. Firstly, integrating Mg^2+^ into the HAp crystal structure allows it to stimulate cell proliferation and differentiation, which can improve bone tissue formation in future applications. Furthermore, due to its chemical properties, HAp-Mg can improve bone biomechanical properties, which is crucial in orthopedic applications. Secondly, incorporating Mg^2+^ into the HAp matrix can enhance the bioactivity and degradation of the material, which favors better integration with host tissues and reduces the risk of implant fallout. Additionally, the antimicrobial activity observed in HAp-Mg 2% is another notable aspect of this study and is especially relevant in orthopedic surgery, where implant-associated infections represent a major concern and can have serious consequences. Finally, the morphology of HAp-Mg nanofibers offers a high surface-to-volume ratio, which facilitates cell adhesion and proliferation, thus promoting tissue regeneration. This study confirms that HAp-Mg nanofibers exhibit both antimicrobial activity and biocompatibility without being cytotoxic or harmful to cells, which is promising for bone regeneration applications. Future research is required to evaluate the in vivo performance of HAp-Mg in bone regeneration and to analyze the long-term effects of Mg^2+^ ion release in host tissues. Finally, HAp-Mg shows significant potential as a biomaterial for bone tissue engineering and the prevention of implant-associated infections, underscoring the importance of continuing to explore its clinical applications and efficacy in vivo models.

## Figures and Tables

**Table 1:** Lattice parameters and average crystallite size were obtained from the Rietveld refinement.

Parameter/Sample	Pure HAp (nm)	HAp-Mg 2% (nm)	HAp-Mg 5% (nm)
a	0.942907 ± 1.8 × 10^5^	0.941681 ± 8.6 × 10^5^	0.94146 ± 1.1 × 10^5^
c	0.688033 ± 4 × 10^5^	0.687138 ± 6.8 × 10^5^	0.685711 ± 8.5 × 10^5^
Crystallite size	96 ± 1.8	55.3 ± 2.6	39.3 ± 1.6

**Table 2:** FTIR band assignments of different samples of HAp.

No.	Mode	Commercial HAp	HAp-Mg 2%	Pure HAp
1	P-O	-	-	411
2	PO_4_^3−^	559	559	559
3	PO_4_^3−^	596	599	599
4	–OH	635	-	635
5	M-O	-	536	-
6	PO_4_^3−^	960	-	962
7	PO_4_^3−^	1018	1021	1021
8	PO_4_^3−^	1098	-	1101

**Table 3:** The element composition of commercial HAp and synthesized HAp-Mg was determined by EDS analysis.

	Commercial HAp		Pure HAp		HAp-Mg 2%		HAp-Mg 5%	
Element	Weight%	Atom%	Weight%	Atom%	Weight%	Atom%	Weight%	Atom%
Carbon	6.061191	10.76036	5.18961	11.78154	1.925941	4.87836	7.076409	12.13933
Oxygen	45.95892	61.26107	39.98652	51.36676	13.29494	25.28091	48.95192	63.04156
Magnesium	-	-	-	-	0.114115	0.142843	0.491616	0.416765
Phosphorus	15.55336	10.71023	18.41139	17.66165	25.85541	25.39612	13.40304	8.916002
Calcium	32.45608	17.27032	41.79752	29.63575	55.5954	42.20295	29.98288	15.41446
Ca/P ratio	-	1.61251	-	1.6779	-	1.66179	-	1.72885

**Table 4:** Diameter of inhibition zones of HAp samples against different types of bacterial and fungal strains.

**HAp-Mg 2% Inhibition Diameter (mm)**									
**Strain**	**Positive Control**	**Standard Error**	Negative Control *	**HAp-Mg 2%** **100 µg/mL**	**Standard Error**	**HAp-Mg 2%** **10 µg/mL**	**Standard Error**	**HAp-Mg 2%** **1 µg/mL**	**Standard Error**
*Escherichia coli*	21.0	0.8	* -	15.0	0.9	12.0	0.7	10.3	1.1
*Enterococcus faecalis*	20.5	0.7	* -	13.5	0.9	11.0	0.6	9.5	0.7
*Staphylococcus aureus*	22.0	1.1	* -	14.0	0.4	9.0	0.9	5.0	0.6
*Candida albicans*	17.0	0.4	* -	8.5	0.6	5.5	0.3	3.5	0.5
**Pure HAp Inhibition Diameter (mm)**									
**Strain**	**Positive Control**	**Standard Error**	Negative Control *	**HAp pure** **100 µg/mL**	**Standard Error**	**HAp pure** **10 µg/mL**	**Standard Error**	**HAp pure** **1 µg/mL**	**Standard Error**
*Escherichia coli*	22.5	1.0	* -	8.5	1.0	7.0	1.2	* -	-
*Enterococcus faecalis*	20.0	1.2	* -	10.0	1.2	7.5	1.0	* -	-
*Staphylococcus aureus*	21.5	0.9	* -	7.2	0.9	6.0	1.5	* -	-
*Candida albicans*	19.0	1.3	* -	6.3	1.3	*-	-	* -	-

* No inhibition zone observed.

## Data Availability

The original contributions presented in the study are included in the article, further inquiries can be directed to the corresponding author.

## References

[1] Bal Z., Kaito T., Korkusuz F., Yoshikawa H. (2020). Bone Regeneration with Hydroxyapatite-Based Biomaterials. Emergent Mater..

[2] Kazimierczak P., Przekora A. (2020). Osteoconductive and Osteoinductive Surface Modifications of Biomaterials for Bone Regeneration: A Concise Review. Coatings.

[3] Samadikuchaksaraei A., Gholipourmalekabadi M., Erfani Ezadyar E., Azami M., Mozafari M., Johari B., Kargozar S., Jameie S., Korourian A., Seifalian A. (2016). Fabrication and in Vivo Evaluation of an Osteoblast-Conditioned Nano-Hydroxyapatite/Gelatin Composite Scaffold for Bone Tissue Regeneration. J. Biomed. Mater. Res. A.

[4] Hu Y., Chen J., Fan T., Zhang Y., Zhao Y., Shi X., Zhang Q. (2017). Biomimetic Mineralized Hierarchical Hybrid Scaffolds Based on in Situ Synthesis of Nano-Hydroxyapatite/Chitosan/Chondroitin Sulfate/Hyaluronic Acid for Bone Tissue Engineering. Colloids Surf. B Biointerfaces.

[5] Ghiasi B., Sefidbakht Y., Mozaffari-Jovin S., Gharehcheloo B., Mehrarya M., Khodadadi A., Rezaei M., Ranaei Siadat S.O., Uskoković V. (2020). Hydroxyapatite as a Biomaterial–a Gift That Keeps on Giving. Drug Dev. Ind. Pharm..

[6] Jing Z., Cao Q., Jun H. (2021). Corrosion, Wear and Biocompatibility of Hydroxyapatite Bio-Functionally Graded Coating on Titanium Alloy Surface Prepared by Laser Cladding. Ceram. Int..

[7] Wang L., Xu L., Peng C., Teng G., Wang Y., Xie X., Wu D. (2019). The Effect of Bone Marrow Mesenchymal Stem Cell and Nano-hydroxyapatite/Collagen I/Poly-L-lactic Acid Scaffold Implantation on the Treatment of Avascular Necrosis of the Femoral Head in Rabbits. Exp. Ther. Med..

[8] dos Santos P.J.P., Yamamura H., Magri A.M.P., Ruiz P.L.M., dos Santos P.J.L., Rennó A.C.M., Ribeiro D.A., Granito R.N. (2021). In Vitro and in Vivo Biological Performance of Hydroxyapatite from Fish Waste. J. Mater. Sci. Mater. Med..

[9] Lu J., Yu H., Chen C. (2018). Biological Properties of Calcium Phosphate Biomaterials for Bone Repair: A Review. RSC Adv..

[10] Lin L., Chow K.L., Leng Y. (2009). Study of Hydroxyapatite Osteoinductivity with an Osteogenic Differentiation of Mesenchymal Stem Cells. J. Biomed. Mater. Res. A.

[11] Zhao X., Yang Z., Liu Q., Yang P., Wang P., Wei S., Liu A., Zhao Z. (2022). Potential Load-Bearing Bone Substitution Material: Carbon-Fiber-Reinforced Magnesium-Doped Hydroxyapatite Composites with Excellent Mechanical Performance and Tailored Biological Properties. CS Biomater. Sci. Eng..

[12] Chen Z., Zhang W., Wang M., Backman L., Chen J. (2022). Effects of Zinc, Magnesium, and Iron Ions on Bone Tissue Engineering. ACS Biomater. Sci. Eng..

[13] Iconaru S.L., Ciobanu C.S., Predoi G., Rokosz K., Chifiriuc M.C., Bleotu C., Stanciu G., Hristu R., Raaen S., Raita S.M. (2022). Biological and Physico-Chemical Properties of Composite Layers Based on Magnesium-Doped Hydroxyapatite in Chitosan Matrix. Micromachines.

[14] Predoi D., Ciobanu C.S., Iconaru S.L., Raaen S., Badea M.L., Rokosz K. (2022). Physicochemical and Biological Evaluation of Chitosan-Coated Magnesium-Doped Hydroxyapatite Composite Layers Obtained by Vacuum Deposition. Coatings.

[15] Predoi D., Iconaru S.L., Predoi M.V., Stan G.E., Buton N. (2019). Synthesis, Characterization, and Antimicrobial Activity of Magnesium-Doped Hydroxyapatite Suspensions. Nanomaterials.

[16] Iconaru S.L., Predoi M.V., Motelica-Heino M., Predoi D., Buton N., Megier C., Stan G.E. (2020). Dextran-Thyme Magnesium-Doped Hydroxyapatite Composite Antimicrobial Coatings. Coatings.

[17] Kumar G.S., Karunakaran G., Girija E.K., Kolesnikov E., Van Minh N., Gorshenkov M.V., Kuznetsov D. (2018). Size and Morphology-Controlled Synthesis of Mesoporous Hydroxyapatite Nanocrystals by Microwave-Assisted Hydrothermal Method. Ceram. Int..

[18] Alanís-Gómez J.R., Rivera-Muñoz E.M., Peza-Ledesma C., Manzano-Ramírez A., Velázquez-Castillo R. (2019). A Comparison of Mechanical Properties of Different Hydroxyapatite (HAp) Based Nanocomposites: The Influence of Morphology and Preferential Orientation. J. Nanosci. Nanotechnol..

[19] Alanís-Gómez J.R., Rivera-Muñoz E.M., Cervantes-Medina J.S., Almanza-Reyes H., Nava-Mendoza R., Cortes-Romero C., Velázquez-Castillo R. (2016). Synthesis of Micro and Nano-Sized Hydroxyapatite Fibers through the Microwave Assisted Hydrothermal Method. J. Nanosci. Nanotechnol..

[20] Alanis-Gómez R.P., Rivera-Muñoz E.M., Luna-Barcenas G., Alanis-Gómez J.R., Velázquez-Castillo R. (2022). Improving the Mechanical Resistance of Hydroxyapatite/Chitosan Composite Materials Made of Nanofibers with Crystalline Preferential Orientation. Materials.

[21] Zhou H., Luchini T.J.F., Bhaduri S.B. (2012). Microwave Assisted Synthesis of Amorphous Magnesium Phosphate Nanospheres. J. Mater. Sci. Mater. Med..

[22] Nabiyouni M., Brückner T., Zhou H., Gbureck U., Bhaduri S.B. (2018). Magnesium-Based Bioceramics in Orthopedic Applications. Acta Biomater..

[23] Zhao S., Jiang Q., Peel S., Wang X., He F. (2013). Effects of Magnesium-Substituted Nanohydroxyapatite Coating on Implant Osseointegration. Clin. Oral. Implants Res..

[24] Zhou H., Liang B., Jiang H., Deng Z., Yu K. (2021). Magnesium-Based Biomaterials as Emerging Agents for Bone Repair and Regeneration: From Mechanism to Application. J. Magnes. Alloys.

[25] Predoi D., Iconaru S.L., Predoi M.V., Motelica-Heino M., Buton N., Megier C. (2020). Obtaining and Characterizing Thin Layers of Magnesium Doped Hydroxyapatite by Dip Coating Procedure. Coatings.

[26] Predoi D., Ciobanu S.C., Iconaru S.L., Predoi M.V. (2023). Influence of the Biological Medium on the Properties of Magnesium Doped Hydroxyapatite Composite Coatings. Coatings.

[27] Hamid R., Rotshteyn Y., Rabadi L., Parikh R., Bullock P. (2004). Comparison of Alamar Blue and MTT Assays for High Through-Put Screening. Toxicol. Vitr..

[28] Kumar P., Nagarajan A., Uchil P.D. (2018). Analysis of Cell Viability by the Alamarblue Assay. Cold Spring Harb. Protoc..

[29] He L.Y., Zhang X.M., Liu B., Tian Y., Ma W.H. (2016). Effect of Magnesium Ion on Human Osteoblast Activity. Braz. J. Med. Biol. Res..

[30] Maier J., Kretsinger R., Uversky V., Permyakov E. (2013). Magnesium and Cell Cycle.

[31] Bodhak S., Bose S., Bandyopadhyay A. (2011). Bone Cell-Material Interactions on Metal-Ion Doped Polarized Hydroxyapatite. Mater. Sci. Eng. C.

[32] Belluci M.M., Schoenmaker T., Rossa-Junior C., Orrico S.R., de Vries T.J., Everts V. (2013). Magnesium Deficiency Results in an Increased Formation of Osteoclasts. J. Nutr. Biochem..

[33] Rude R.K., Wei L., James Norton H., Lu S.S., Dempster D.W., Gruber H.E. (2009). TNFα Receptor Knockout in Mice Reduces Adverse Effects of Magnesium Deficiency on Bone. Growth Factors.

[34] Nguyen N.Y.T., Grelling N., Wetteland C.L., Rosario R., Liu H. (2018). Antimicrobial Activities and Mechanisms of Magnesium Oxide Nanoparticles (NMgO) against Pathogenic Bacteria, Yeasts, and Biofilms. Sci. Rep..

[35] Demishtein K., Reifen R., Shemesh M. (2019). Antimicrobial Properties of Magnesium Open Opportunities to Develop Healthier Food. Nutrients.

[36] Nagaraj A., Samiappan S. (2019). Presentation of Antibacterial and Therapeutic Anti-Inflammatory Potentials to Hydroxyapatite via Biomimetic With Azadirachta Indica: An in Vitro Anti-Inflammatory Assessment in Contradiction of LPS-Induced Stress in RAW 264.7 Cells. Front. Microbiol..

[37] Nagaraj S., Manivannan S., Narayan S. (2021). Potent Antifungal Agents and Use of Nanocarriers to Improve Delivery to the Infected Site: A Systematic Review. J. Basic. Microbiol..

[38] Tornero-Gutiérrez F., Ortiz-Ramírez J.A., López-Romero E., Cuéllar-Cruz M. (2023). Materials Used to Prevent Adhesion, Growth, and Biofilm Formation of Candida Species. Med. Mycol..

